# Miss Matheson Speaks at Belfast

**Published:** 1917-08-04

**Authors:** 


### THE COLLEGE OF NURSING, LIMITED.

### Miss Matheson Speaks at Belfast.

A REMARKABLE meeting in connection with the College of Nursing was held at the Medical Institute, Belfast, on the 26th ultimo. Thanks largely to the administrative gifts of the local Hon. Secretary (Miss Curtin, matron of the Mater Infirmorum Hospital), over seven hundred nurses and doctors were invited to take an interest in the College, and there were 200 nurses and doctors present, under the presidency of Sir Alexander Dempsey. Sir Alexander defined the main object of the College to be to elevate the nursing profession and give it a legal status such as that enjoyed by the medical and dental professions. He was sure the College would have the sympathy and support of the public in its endeavour to promote the advancement of nurses, who were not only hard-working, but devoted women workers.

Miss Matheson, Secretary of the Irish Board of the College of Nursing, Limited, gave an exhaustive address, and displayed great ability and a complete mastery of her subject. "For very many years, as you all know," she declared, "there have been in existence in England, Scotland, and Ireland a great many hospitals, large and small, professing to train nurses. In some the training is good, in some it is indifferent, in some it is actually bad. Some offer two, some three, some four years' training. In some probationers spend their whole time in the wards or departments of the hospital; in others they spend a good deal in going out to private cases. In some the probationers have no responsibility till after the first year, or even two; in some they are thrust into it in a few weeks or months. It follows that at present the trained nurse may mean anything from the very highest expression in knowledge, skill, discipline, gentleness, and sympathy, or she may mean—what too many who have been unlucky think all nurses embody—carelessness, ignorance, clumsiness, and roughness. The present state of affairs is unfair both to the public and the nurses. The profession has lacked organisation, though there are in existence so-called self-governing associations, large and small, many of which have been more or less moribund all their lives, and some of which have done good work. There is no co-ordination amongst them or co-operation in effective work, and nurses as a body, whatever their aspirations, have had no means of voicing their desires.

### STATE REGISTRATION.

Such has been more or less the state of affairs for many years. Now I want to say something here about State Registration. For upwards of twenty years Mrs. Bedford Fenwick, the editor of the *British Journal of Nursing*, has given time and untiring energy in the endeavour to promote the State Registration of trained nurses. She has done much in an endeavour to educate public opinion in this matter. She is convinced, and has convinced others, that once nurses are registered by the State everything else will follow, and that nothing can be done until they are State Registered, just as a great many suffragists believe that the vote will bring the millennium, and that nothing can be done without the vote. But if the women of Great Britain and Ireland had sat down and said 'We can do nothing for our fellow-women until we get the vote,' I think we'd all have been in rather a bad way. Mrs. Bedford Fenwick and her Committee, and those Associations which think with her, have made no attempt, beyond trying to educate public opinion in favour of State Registration, to combine all forces and *do* something for the nursing profession whilst waiting for their Bill to be favourably received in Parliament. Had she devoted some of her splendid energy to endeavouring to co-operate with other forces to bring about reform within the profession itself, I imagine there would not have been the bitterness and disunion on this question which has made any advance so difficult.

### AN OUTCOME OF THE WAR.

Such was the position when the war came, upsetting all preconceived notions about everything, and demanding, amongst other things, nurses, nurses, and still more nurses. The trained nurses responded gallantly, and they were seconded by the members of the British Red Cross and St. John Ambulance Association. Now these Associations have a Joint War Committee, and at the head of that Committee, as its Chairman, is the Hon. Arthur Stanley, C.B., M.P. Brought thus into contact with the nursing profession, he was struck very much by the condition of things which I have described to you as obtaining at that time. And he felt that surely something could be done. So he talked to various people who he considered would know something about the matter, and he found that they quite agreed with him. So then he had the inspiration of founding a College of Nursing, which would do for nursing what other professions had had done for them by Chartered Institutes, Royal Colleges, and suchlike. I don't know what you think, but to me the very words 'College of Nursing' sound more inspiring than any number of societies or associations. They invoke a mental picture. Because our profession is one of service and embodies some of the highest ideas of humanity, those of us who think at all must feel—even if we have not time to think it out or formulate it—that it should have some outward expression, apart from our own work as individuals, worthy of those ideas. Some of us find it in our training-schools, but hospitals are obliged to devote their money primarily to their patients, and there is seldom any surplus. What we want is a universal concrete expression of the ideals which are at the heart of our profession. And I most earnestly hope that in the course of time we shall see in London a building which shall be the centre of nursing education, where examinations will be held, where there will be special lecturers—not only medical men, but specially trained nurses giving free courses of lectures—where nurses can enter for special scholarships and a multiplicity of other desirable things, where everything will be of the very best, and where there will be up-to-date professional libraries of the newest books and the latest ideas, where all the nursing papers can be read and information and advice obtained about everything connected with nursing. And I believe also that in that bald sentence, 'To establish centres in different parts of the kingdom,' lie great possibilities not only for the large provincial towns of England, but for Belfast and Dublin and Cork, and any other centre where nurses are anxious to make the most of their opportunities and to co-operate for the good of all.

### 'HANDED TO THE NURSES TO RUN THEMSELVES.'

Having conceived the idea of a College, Mr. Stanley saw, however, that there was so much difference about State Registration that the College would have to be at first a voluntary body with a voluntary Register, and that the only way of enabling nurses to have, like other professions, a democratic governing body elected by themselves and fully representing them, was to enlist the services of those of their profession who by their position and experience had the time and the knowledge to set up the machinery of the College. Once established firmly on its feet it would be handed over to the nurses to run themselves. He hoped that in the College might be co-ordinated the efforts of the various associations working on different lines for the betterment of the profession. And so the College of Nursing was founded. If you look at what the College has been doing during the past year, you will see that it is working first and foremost for the trained nurse. Some of you know that in September of last year the War Office appointed a Committee to inquire into the supply of trained nurses for the Army. As that Committee

(Continued on page iv.)

